# Supplementary material for: HIVprotI: an integrated web based platform for prediction and design of HIV proteins inhibitors
Source: J Cheminform. 2018 Mar 9;10:12. doi: 10.1186/s13321-018-0266-y (PMC5845081; doi:10.1186/s13321-018-0266-y)
Supplement: Supplementary file 2 — Additional file 2. Source code of HIVProtI web server. [file 13321_2018_266_MOESM2_ESM.zip › HIVprotI_Source-code/hivprotI/weka-3-6-6/doc/weka/experiment/InstancesResultListener.html]

InstancesResultListener


---


|  |  |  |
| --- | --- | --- |
| **Overview** \| **Package** \| **Class** \| **Tree** \| **Deprecated** \| **Index** \| **Help** \| **Weka's home** | |  |
| **PREV CLASS**   **NEXT CLASS** | **FRAMES**    **NO FRAMES**     **All Classes** |  |
| SUMMARY: NESTED \| FIELD \| CONSTR \| METHOD | DETAIL: FIELD \| CONSTR \| METHOD |  |


---


## weka.experiment Class InstancesResultListener

```
java.lang.Object
  weka.experiment.CSVResultListener
      weka.experiment.InstancesResultListener
```

**All Implemented Interfaces:**: java.io.Serializable, OptionHandler, RevisionHandler, ResultListener

---

``` public class InstancesResultListener extends CSVResultListener ```

Outputs the received results in arff format to a Writer. All results must be received before the instances can be written out.


Valid options are:

```
 -O <file name>
  The filename where output will be stored. Use - for stdout.
  (default temp file)
```

**Version:**
:   $Revision: 1.11 $

**Author:**
:   Len Trigg (trigg@cs.waikato.ac.nz)

**See Also:**: Serialized Form

---

**Constructor Summary**

`InstancesResultListener()`
  
          Sets temporary file.


| **Method Summary** | |
| --- | --- |
| `void` | `acceptResult(ResultProducer rp, java.lang.Object[] key, java.lang.Object[] result)`             Collects each instance and adjusts the header information. |
| `java.lang.String` | `getRevision()`             Returns the revision string. |
| `java.lang.String` | `globalInfo()`             Returns a string describing this result listener |
| `void` | `postProcess(ResultProducer rp)`             Perform any postprocessing. |
| `void` | `preProcess(ResultProducer rp)`             Prepare for the results to be received. |

**Methods inherited from class weka.experiment.CSVResultListener**

`determineColumnConstraints, getOptions, getOutputFile, isResultRequired, listOptions, outputFileName, outputFileTipText, setOptions, setOutputFile, setOutputFileName`

**Methods inherited from class java.lang.Object**

`equals, getClass, hashCode, notify, notifyAll, toString, wait, wait, wait`

**Constructor Detail**

### InstancesResultListener

```
public InstancesResultListener()
```

:   Sets temporary file.


**Method Detail**

### globalInfo

```
public java.lang.String globalInfo()
```

:   Returns a string describing this result listener

    :   **Overrides:**: `globalInfo` in class `CSVResultListener`
    :   **Returns:**: a description of the result listener suitable for displaying in the explorer/experimenter gui

---


### preProcess

```
public void preProcess(ResultProducer rp)
                throws java.lang.Exception
```

:   Prepare for the results to be received.

    :   **Specified by:**: `preProcess` in interface `ResultListener` **Overrides:**: `preProcess` in class `CSVResultListener`
    :   **Parameters:**: `rp` - the ResultProducer that will generate the results **Throws:**: `java.lang.Exception` - if an error occurs during preprocessing.

---


### postProcess

```
public void postProcess(ResultProducer rp)
                 throws java.lang.Exception
```

:   Perform any postprocessing. When this method is called, it indicates
    that no more results will be sent that need to be grouped together
    in any way.

    :   **Specified by:**: `postProcess` in interface `ResultListener` **Overrides:**: `postProcess` in class `CSVResultListener`
    :   **Parameters:**: `rp` - the ResultProducer that generated the results **Throws:**: `java.lang.Exception` - if an error occurs

---


### acceptResult

```
public void acceptResult(ResultProducer rp,
                         java.lang.Object[] key,
                         java.lang.Object[] result)
                  throws java.lang.Exception
```

:   Collects each instance and adjusts the header information.

    :   **Specified by:**: `acceptResult` in interface `ResultListener` **Overrides:**: `acceptResult` in class `CSVResultListener`
    :   **Parameters:**: `rp` - the ResultProducer that generated the result: `key` - The key for the results.: `result` - The actual results. **Throws:**: `java.lang.Exception` - if the result could not be accepted.

---


### getRevision

```
public java.lang.String getRevision()
```

:   Returns the revision string.

    :   **Specified by:**: `getRevision` in interface `RevisionHandler` **Overrides:**: `getRevision` in class `CSVResultListener`
    :   **Returns:**: the revision


---


|  |  |  |
| --- | --- | --- |
| **Overview** \| **Package** \| **Class** \| **Tree** \| **Deprecated** \| **Index** \| **Help** \| **Weka's home** | |  |
| **PREV CLASS**   **NEXT CLASS** | **FRAMES**    **NO FRAMES**     **All Classes** |  |
| SUMMARY: NESTED \| FIELD \| CONSTR \| METHOD | DETAIL: FIELD \| CONSTR \| METHOD |  |


---
